# Accuracy of Lumbar Pedicle Screw Placement Based on Insertion Techniques in a Single-Center Retrospective Study

**DOI:** 10.7759/cureus.93528

**Published:** 2025-09-29

**Authors:** Takanobu Miyamoto, Nobuaki Naito, Hokuto Yamashita, Shigeo Ueda, Jiro Ohara

**Affiliations:** 1 Shin-ai Kai Spine Center, Katano Hospital, Katano, JPN

**Keywords:** fluoroscopy, image-guided surgery, lumbar vertebrae, pedicle screws, surgical navigation systems

## Abstract

Introduction: Although many studies have evaluated the accuracy of pedicle screw (PS) placement using various techniques, few have conducted large-scale comparisons of open and percutaneous approaches using CT navigation and fluoroscopy within a single institution. This study aimed to evaluate the accuracy of lumbar PS placement based on four different insertion techniques and to identify factors influencing screw placement accuracy.

Methods: A total of 237 cases (1,081 screws) of PS placement at the L1-S1 level were retrospectively analyzed. Patients were divided into four groups: open CT navigation, percutaneous PS (PPS) CT navigation, PPS fluoroscopy, and open freehand. Screw accuracy was assessed using the Gertzbein-Robbins classification based on postoperative CT or intraoperative O-arm images. The proportions of grade A (no breach) and grade A or B (≤2 mm breach) screws were compared among the groups. Statistical analyses included Fisher’s exact test with Bonferroni correction.

Results: The proportion of grade A screws was highest in the open CT navigation group (96.4%), followed by PPS fluoroscopy (89.3%), open freehand (88.0%), and PPS CT navigation (81.0%) (p < 0.05). Grade A or B screws exceeded 95% in all groups with no significant differences. Lateral breaches were the most frequent, particularly in the PPS CT navigation (93.3%) and PPS fluoroscopy (82.0%) groups. No grade D or E medial or caudal breaches or neurological complications were observed. Screw repositioning was required in two cases (three screws) due to lateral breaches >4 mm with screw tips located outside the vertebral body.

Conclusions: All four insertion techniques achieved high accuracy, with more than 95% of screws classified as acceptable placements and no neurological complications observed. These findings suggest that lumbar PS placement can be performed safely and reliably across different techniques when applied appropriately.

## Introduction

Pedicle screws (PS) were first introduced by Boucher in 1959 [1] and are now widely used for lumbar spinal procedures, including lumbar interbody fusion (LIF), posterolateral fusion (PLF), and vertebral body replacement. PS provide strong biomechanical fixation and have been associated with favorable clinical outcomes. However, improper placement carries a risk of serious complications such as neural or vascular injury.

Regarding the accuracy of PS placement, a systematic review by Gelalis et al. [2] and a meta-analysis by Perdomo-Pantoja et al. [3] demonstrated that the breach rate with CT navigation is significantly lower than that with conventional fluoroscopy or freehand methods. In particular, the use of CT navigation has been widely adopted at the cervical and thoracic levels, where pedicles are narrow and the risk of spinal cord injury due to medial breaches is substantial.

Recently, the adoption of minimally invasive surgery (MIS) has increased the use of percutaneous pedicle screws (PPS), which offer advantages such as reduced blood loss and decreased soft tissue trauma compared to traditional open procedures [4]. However, PPS placement requires advanced surgical skill due to the lack of direct visual confirmation of anatomical landmarks.

At our center, all PS placements at the cervical and thoracic levels are performed under CT navigation guidance. In contrast, multiple insertion techniques are used at the lumbar level, including open and PPS approaches, with guidance via CT navigation, fluoroscopy, or no guidance (freehand). While previous studies have compared the accuracy of these techniques [5-7], few have conducted large-scale comparisons within a single institution.

The purpose of this study was to compare the accuracy of lumbar PS placement among insertion techniques used at our center and to identify factors that may influence placement accuracy. This study was designed as an exploratory, hypothesis-generating analysis rather than a hypothesis-testing trial.

## Materials and methods

Ethics statement

This study was performed in accordance with the Helsinki Declaration and was approved by the Institutional Review Board of Katano Hospital (approval number: 20240517-2). Written informed consent was waived due to the retrospective nature of the study.

Study design and patient selection

This retrospective study included patients who underwent PS placement at the L1-S1 level at our institution between January 2022 and December 2023. Screws inserted using pre-existing screw holes during revision surgery or cases in which all screws were replacements were excluded.

Surgical techniques

Patients were divided into four groups based on the surgical technique. The first group underwent PS insertion via an open approach using an intraoperative O-arm CT scan (Medtronic, Minneapolis, MN) and StealthStation S7 navigation system (Medtronic); this group was defined as the open CT navigation (Open CT-nav) group. The second group underwent percutaneous PS insertion under the same CT navigation guidance; this group was defined as the PPS CT navigation (PPS CT-nav) group. The third group underwent percutaneous PS insertion under biplanar fluoroscopic guidance using two C-arm units in anteroposterior and lateral views; this group was defined as the PPS fluoroscopy (PPS Fluoro) group. The fourth group underwent PS insertion via an open approach without navigation guidance; this group was defined as the open freehand group. In the open approach, after midline skin incision, paraspinal muscles were dissected to expose the lamina and facet joints. The entry point for PS was located on the superior articular process, and the trajectory followed the anatomical axis of the pedicle. In the PPS approach, a 3 cm transverse or longitudinal skin incision for each screw was made approximately 5 cm lateral to the midline. After incising the fascia, blunt dissection was performed manually to palpate the base of the transverse process, which was used as the entry point for PS placement.

All procedures were performed by board-certified spine surgeons. In the Open CT-nav group, surgeons A (24 years post-graduation), B (24 years), C (18 years), and D (16 years) performed the surgeries. The PPS CT-nav group included surgeons A, C, D, and E (10 years). The PPS Fluoro group included surgeons A and F (22 years). The open freehand group included surgeons B and C. Thus, six surgeons in total participated, with post-graduate experience ranging from 10 to 24 years.

Assessment of screw accuracy

The evaluation of screw breach distance and direction was performed by a single spine surgeon who was not involved in the surgeries. Although the evaluator was blinded to patient information, complete blinding to the insertion technique was not possible, as the approach (open vs. percutaneous) could often be recognized on postoperative CT or intraoperative O-arm images. The breach distance was assessed using the Gertzbein-Robbins classification (grade A: no breach; grade B: breach ≤ 2 mm; grade C: breach = 2-4 mm; grade D: breach = 4-6 mm; grade E: breach > 6 mm) [8], and the breach direction was categorized as lateral, medial, cranial, or caudal. Screw placement accuracy was assessed by calculating the proportion of screws classified as grade A (“perfect insertion accuracy”) and the proportion of screws classified as grade A or B (“acceptable insertion accuracy”).

Statistical analysis

For statistical analysis, Fisher’s exact test was used to compare proportions among the four groups, and the Bonferroni correction was applied to adjust for multiple comparisons. Continuous variables in patient backgrounds were analyzed using one-way analysis of variance (ANOVA). A p-value of < 0.05 was considered statistically significant. All analyses were performed using Python version 3.11 (Python Software Foundation, Wilmington, Delaware) with the scipy and statsmodels packages.

## Results

A total of 240 cases (1,117 screws) were identified, of which three cases (36 screws) were excluded based on the criteria described. Thus, 237 cases with 1,081 screws were included in the analysis. The group distribution was as follows: Open CT-nav group: 45 cases (247 screws); PPS CT-nav group: 35 cases (158 screws); PPS Fluoro group: 127 cases (568 screws); open freehand group: 27 cases (108 screws).

Patient demographics are summarized in Table 1. There were no significant differences in age, height, weight, or BMI among the four groups. The most common surgical indication was degenerative lumbar disease, and the most frequently performed procedure was posterior lumbar interbody fusion (PLIF) in all groups. Notably, all cases in the open freehand group underwent PLIF. The most common screw insertion levels were L4 and L5 in all groups, and most screws had an outer diameter of 6.5 mm (Table 2).

**Table 1:** Patient demographics. Age, sex, height, weight, and body mass index (BMI) for each group. Values are presented as mean ± standard deviation or percentage. P-values were calculated using one-way analysis of variance (ANOVA) for continuous variables and Fisher’s exact test for categorical variables. PPS, percutaneous pedicle screw.

Variable	Open CT-nav (n = 45)	PPS CT-nav (n = 35)	PPS Fluoro (n = 127)	Open freehand (n = 27)	P-value
Age (years)	71.9 ± 8.5	68.8 ± 12.9	69.2 ± 10.4	71.5 ± 10.7	0.357
Male	19 (42.2)	16 (45.7)	54 (42.5)	10 (37.0)	0.924
Height (cm)	156.9 ± 11.1	158.4 ± 9.5	157.4 ± 9.5	158.6 ± 8.5	0.868
Weight (kg)	58.2 ± 14.1	60.1 ± 11.8	59.6 ± 9.8	61.2 ± 11.5	0.713
BMI (kg/m^2^)	23.5 ± 4.4	23.9 ± 4.0	24.0 ± 3.2	24.2 ± 3.1	0.824

**Table 2:** Types of surgery and screw characteristics. Types of surgical procedures, number of screws placed at each vertebral level (L1–S1), and screw diameters for each group. “Other LIF” includes TLIF, OLIF, and XLIF. PLIF, posterior lumbar interbody fusion; TLIF, transforaminal lumbar interbody fusion; OLIF, oblique lateral interbody fusion; XLIF, extreme lateral interbody fusion; PLF, posterolateral fusion; LIF, lumbar interbody fusion; PPS, percutaneous pedicle screw.

Variable	Open CT-nav (n = 45 patients, 247 screws)	PPS CT-nav (n = 35 patients, 158 screws)	PPS Fluoro (n = 127 patients, 568 screws)	Open freehand (n = 27 patients, 108 screws)
Type of surgery				
PLIF	30	20	87	27
Other LIF	5	9	37	0
PLIF + Other LIF	3	0	0	0
PLF	7	5	2	0
Others	0	1	1	0
Number of screws				
L1	12	12	10	0
L2	24	26	24	2
L3	42	20	68	16
L4	50	40	172	44
L5	69	44	208	38
S1	50	16	86	8
Screw diameter				
4.5 mm	0	2	0	0
5.5 mm	23	16	6	2
6.0 mm	17	10	130	4
6.5 mm	181	124	417	102
7.0 mm	0	6	15	0
7.5 mm	26	0	0	0

The proportion of screws classified as grade A was as follows: Open CT-nav: 96.4% (238/247); PPS CT-nav: 81.0% (128/158); PPS Fluoro: 89.3% (507/568); open freehand: 88.0% (95/108). Multiple comparisons revealed that the Open CT-nav group had a significantly higher proportion of grade A screws than the PPS CT-nav (p < 0.001), PPS Fluoro (p = 0.004), and open freehand (p = 0.0403) groups. In contrast, the proportion of screws classified as grade A or B was 98.4%, 96.2%, 96.7%, and 95.4%, respectively, with no significant differences among the four groups (Table 3).

**Table 3:** Accuracy of pedicle screw placement. The proportion of screws classified as grade A and grade A or B, according to the Gertzbein-Robbins classification, is shown for each group. Results are presented for all levels (L1–S1) and for L4–5 levels only. P-values indicate the results of Fisher’s exact test with Bonferroni correction for multiple comparisons. Group comparisons: (1) Open CT-nav; (2) PPS CT-nav; (3) PPS Fluoro; (4) Open Freehand. G-R classification, Gertzbein-Robbins classification; PPS, percutaneous pedicle screw.

G-R classification	Level	Open CT-nav	PPS CT-nav	PPS Fluoro	Open freehand	P-value					
						(1) vs. (2)	(1) vs. (3)	(1) vs. (4)	(2) vs. (3)	(2) vs. (4)	(3) vs. (4)
Grade A	L1-S1	238 (96.4)	128 (81.0)	507 (89.3)	95 (88.0)	<0.001	0.004	0.0403	0.056	1.0	1.0
	L4-5	115 (96.6)	67 (79.8)	73 (89.0)	350 (91.1)	<0.001	0.289	0.246	0.034	0.808	1.0
Grade A or B	L1-S1	243 (98.4)	152 (96.2)	549 (96.7)	103 (95.4)	1.0	1.0	0.122	1.0	1.0	0.932
	L4-5	118 (99.2)	82 (97.6)	373 (97.1)	77 (93.9)	1.0	1.0	0.254	1.0	1.0	1.0

In an exploratory analysis limited to the L4 and L5 levels, the proportion of grade A screws was significantly higher in both the Open CT-nav and PPS Fluoro groups than in the PPS CT-nav group (p < 0.01 and p = 0.034, respectively). However, the grade A or B proportions remained statistically comparable across all groups in this analysis as well.

Regarding the direction of breaches, lateral breaches were the most frequent in all groups: Open CT-nav: 66.7% (6/9); PPS CT-nav: 93.3% (28/30); PPS Fluoro: 82.0% (50/61); open freehand: 69.2% (9/13). PPS CT-nav and PPS Fluoro groups had particularly high rates of lateral breaches (Table 4). No grade D or E breaches in the medial or inferior direction were observed in any group.

**Table 4:** Screw breach directions. Distribution of screw breach directions (lateral, medial, cranial, caudal) for screws with breaches in each group. Values are presented as numbers (%). PPS, percutaneous pedicle screw.

Direction	Open CT-nav (n = 9)	PPS CT-nav (n = 30)	PPS Fluoro (n = 61)	Open freehand (n = 13)
Lateral	6 (66.7)	28 (93.3)	50 (82)	9 (69.2)
Medial	0	2 (6.7)	7 (11.5)	3 (23.1)
Cranial	1 (11.1)	0	2 (3.3)	0
Caudal	2 (22.2)	0	2 (3.3)	1 (7.7)

No neurological or dural complications associated with pedicle wall breaches were observed. In two cases (three screws), where intraoperative O-arm imaging revealed lateral breaches of >4 mm with screw tips located outside the vertebral body, intraoperative screw repositioning was performed.

## Discussion

Gertzbein et al. classified pedicle screw accuracy according to the degree of cortical breach, and their grading system is widely used today [8]. A breach greater than 4 mm (grade D or E) extending medially into the spinal canal or caudally into the foramen has been reported to carry a high risk of neurological injury [8-10]. Therefore, many studies have defined acceptable accuracy as a breach of within 2 mm (grade A or B) or within 4 mm (up to grade C). In our study, no breaches exceeding 4 mm in the medial or caudal direction were observed, and notably, none of the patients with breaches within 4 mm in these directions developed neurological deficits.

Gelalis et al. (2012, systematic review) [2] reported that the proportion of screws with a within 2 mm breach was 89-100% for CT navigation, 28-85% for fluoroscopy, and 69-94% for the freehand technique. Perdomo-Pantoja et al. (2019, meta-analysis) [3] reported that the proportion of pedicle screws with less than 4 mm breach was 95.5% for CT navigation, 91.5% for fluoroscopy, and 93.1% for the freehand technique. Both reviews consistently demonstrated the superiority of CT navigation over other techniques. In our study, the acceptable insertion accuracy was 98.4% for the Open CT-nav group, 96.2% for the PPS CT-nav group, 96.7% for the PPS Fluoro group, and 95.4% for the open freehand group, all exceeding 95%. Although no statistically significant differences were observed among the techniques, the trend was consistent with previous reports, with CT navigation showing the highest accuracy, followed by fluoroscopy and the freehand technique. The high accuracy in our study may be attributable to the fact that our cohort included only lumbosacral levels. The two aforementioned studies targeted thoracolumbar levels. Perdomo-Pantoja et al. reported that studies with a higher proportion of thoracic screws tended to show lower accuracy [3]. The lower accuracy at thoracic levels has been linked to their smaller pedicle width compared with lumbar levels, which leaves less margin for screw containment. By contrast, the higher accuracy in our study likely reflects the predominance of lower lumbar levels. In this region, pedicles are wider than at upper lumbar levels [11].

In addition to the fact that this study was limited to lumbar levels, the exclusive use of PLIF procedures in the open freehand group may have contributed to the improved accuracy. All cases in this group underwent PLIF, and at our institution, the PLIF technique includes resection of the inferior articular process of the upper vertebra and the superior articular process of the lower vertebra before disc space access. This provides a clear view of the medial and superior/inferior pedicle borders, allowing direct three-dimensional visualization of pedicle anatomy, which may reduce the risk of medial breach under direct vision. Salama et al. [12] similarly reported that direct visualization of pedicle and disc-space landmarks facilitated safe freehand screw insertion with a low breach rate. Consistent with these findings, our results suggest that freehand PS placement during PLIF can achieve favorable accuracy and safety.

A key finding of the study was the significant difference in perfect insertion accuracy between the Open CT-nav group (grade A: 96.4%) and the PPS CT-nav group (grade A: 81.0%), despite the use of the same navigation system. We believe this difference can be attributed to two key technical factors specific to the PPS technique. First, the entry point and trajectory in PPS are different. PPS are generally inserted at the lateral base of the transverse process in a more oblique trajectory, often diverging from the anatomical axis of the pedicle. This may lead to a higher likelihood of lateral breach. In contrast, in open procedures, the entry point is typically on the superior articular process, closer to the pedicle axis, which allows for more aligned insertion. The high incidence of lateral breaches in the PPS CT-nav (93.3%) and PPS Fluoro (82.0%) groups supports this anatomical and technical explanation. Second, the limited ability to detect skiving (slippage of the screw over the bony surface) in PPS procedures may also reduce accuracy. In open procedures, the cortical bone surface can be directly observed and modified as needed. In contrast, PPS does not permit direct visualization of the bone surface. In cases of sclerotic bone or sloped surfaces, skiving is more likely to occur, and CT navigation, which relies on sliced images, may not detect subtle deviations. The PPS Fluoro group showed slightly better accuracy than the PPS CT-nav group. One possible explanation, generated from our data, is that real-time fluoroscopy allows continuous visualization of screw advancement, which might facilitate earlier detection of skiving. However, this remains a hypothesis and should be interpreted cautiously rather than as a definitive conclusion.

Several limitations should be acknowledged. First, this was a retrospective, non-randomized study, which introduces the potential for selection and confounding bias. In particular, the open freehand group exclusively consisted of PLIF cases, and this procedural difference may have contributed to the higher accuracy observed in this group, representing a key confounder. Second, although the study included a relatively large total number of screws, the sample sizes of three of the four groups were modest, which may have limited statistical power. Third, procedures were performed by six board-certified spine surgeons with varying levels of post-graduate experience (10-24 years). Although this reflects real-world clinical practice, it introduces potential variability in technique-related accuracy, which was not controlled for in the analysis. Fourth, we did not perform detailed morphometric measurements of pedicle anatomy or screw insertion angles. Consequently, our explanation regarding the high rate of lateral breaches in the PPS groups remains speculative and warrants confirmation through quantitative trajectory analysis. Finally, while significant differences were observed in “perfect” accuracy (grade A), all groups achieved high rates of “acceptable” accuracy (grade A or B), and no neurological complications occurred. This raises questions about the clinical significance of striving for perfect accuracy. Additionally, further biomechanical studies are required to clarify whether lateral breaches influence long-term pedicle screw fixation strength.

## Conclusions

In this retrospective single-center study, we compared the accuracy of lumbar pedicle screw placement using four insertion techniques. All groups achieved high accuracy, with more than 95% of screws classified as acceptable insertions, and no breaches exceeding 4 mm in the medial or caudal direction were observed. No patients developed neurological deficits attributable to screw malposition. These findings indicate that lumbar pedicle screw placement can be performed safely and reliably across different techniques when applied appropriately.
